# Plasma Technology Increases the Efficacy of Prothioconazole against *Fusarium graminearum* and *Fusarium proliferatum* Contamination of Maize (*Zea mays*) Seedlings

**DOI:** 10.3390/ijms22179301

**Published:** 2021-08-27

**Authors:** Mario Masiello, Stefania Somma, Chiara Lo Porto, Fabio Palumbo, Pietro Favia, Francesco Fracassi, Antonio Francesco Logrieco, Antonio Moretti

**Affiliations:** 1 Institute of Sciences of Food Production (ISPA), National Research Council of Italy, Via Amendola 122/O, 70126 Bari, Italy; mario.masiello@ispa.cnr.it (M.M.); stefania.somma@ispa.cnr.it (S.S.); antonio.logrieco@ispa.cnr.it (A.F.L.); 2 Department of Chemistry, University of Bari Aldo Moro, Via Orabona 4, 70126 Bari, Italy; c.loporto@ba.ipcf.cnr.it (C.L.P.); pietro.favia@uniba.it (P.F.); francesco.fracassi@uniba.it (F.F.); 3 Institute of Nanotechnology, National Research Council of Italy, c/o Department of Chemistry, University of Bari Aldo Moro, Via Orabona 4, 70126 Bari, Italy; fabio.palumbo@cnr.it

**Keywords:** eco-friendly strategies, germinability, mycotoxins, systemic transmission

## Abstract

The contamination of maize by *Fusarium* species able to produce mycotoxins raises great concern worldwide since they can accumulate these toxic metabolites in field crop products. Furthermore, little information exists today on the ability of *Fusarium proliferatum* and *Fusarium graminearum*, two well know mycotoxigenic species, to translocate from the seeds to the plants up to the kernels. Marketing seeds coated with fungicide molecules is a common practice; however, since there is a growing need for reducing chemicals in agriculture, new eco-friendly strategies are increasingly tested. Technologies based on ionized gases, known as plasmas, have been used for decades, with newer material surfaces, products, and approaches developed continuously. In this research, we tested a plasma-generated bilayer coating for encapsulating prothioconazole at the surface of maize seeds, to protect them from *F. graminearum* and *F. proliferatum* infection. A minimum amount of chemical was used, in direct contact with the seeds, with no dispersion in the soil. The ability of *F. graminearum* and *F. proliferatum* species to translocate from seeds to seedlings of maize has been clearly proven in our in vitro experiments. As for the use of plasma technology, the combined use of the plasma-generated coating with embedded prothioconazole was the most efficient approach, with a higher reduction of the infection of the maize seminal root system and stems. The debated capability of the two *Fusarium* species to translocate from seeds to seedlings has been demonstrated. The plasma-generated coating with embedded prothioconazole resulted in a promising sustainable approach for the protection of maize seedlings.

## 1. Introduction

Maize is one of the most cultivated crops worldwide, and a staple food for human and livestock in several geographical areas. In addition, environmentally sustainable policies have led to the proposition of new maize by-products, such as starch, fibers, and oil in the food and chemical industry [1,2].

Maize can be colonized by several fungal pathogens, among which species belonging to the *Fusarium* genus are of particular concern. Many of them, particularly the soil and seed-borne pathogens, are associated with some of the most devastating fungal diseases of maize, such as Fusarium ear and stalk rots, Fusarium stem and root rots, and seedling decay, and cause significant losses of products all over the world. In addition, many *Fusarium* species produce a wide range of mycotoxins—harmful secondary metabolites that accumulate in plants throughout their growth. The seedling stage, flowering time, and wax ripe stage of kernels are all highly susceptible periods during which maize plants can be colonized by *Fusarium* species [3]. For this reason, maize intended for food and feed results in frequently being contaminated by *Fusarium* mycotoxins, as well as the maize-based by-products, since these mycotoxins often remain stable also at the high temperature used in food processing [4]. Fumonisins (FBs), deoxynivalenol (DON), and zearalenone (ZEA) are the most important mycotoxins accumulated by *Fusarium* species in both stalks and kernels, at harvest, and in the field [4]. 

While FBs are mainly produced by *F. verticillioides* and *F. proliferatum*, *F. graminearum* is the main species responsible for the production of DON and ZEA [4]. These species can infect maize plants through the root system from the soil, from contaminated seeds, or through spores carried over by wind, water, and insect wounds. The ability of *F. verticillioides* to systemically colonize seedlings from seeds is well known and has been deeply investigated by several authors for decades [5,6,7]. On the other hand, the knowledge of the transmission path of *F. graminearum* and *F. proliferatum* from seeds to seedlings is poor and controversial, although some papers report that the latter species shares a similar disease cycle with *F. verticillioides* [8]. Indeed, *F. proliferatum* and *F. verticillioides* have been often reported as associated in colonizing maize plants worldwide [8,9,10,11,12]. *F. proliferatum* is a ubiquitous species, able to colonize a wide range of important agricultural crops besides maize and other cereals, such as vegetables and fruit trees [13]. Moreover, *F. proliferatum* produces also other mycotoxins, such as moniliformin [14], beauvericin [15], fusaproliferin [16], and fusarins [17], thus increasing the risk of multiple mycotoxin occurrence in foodstuff. Besides the abovementioned species, *F. graminearum* has been reported as a maize pathogen in geographical areas where the environmental conditions are colder or when the maize is cultivated as a second crop and is exposed to low temperatures and high humidity [18]. According to the single species, *Fusarium* infections occurring in the first growth stages of maize can contribute to the final mycotoxin contamination in kernels, since this early colonization of plant tissues is usually followed by the further progress of the infection up to the ears at harvest, in certain environmental conditions [19]. Therefore, coating the seeds with fungicides and/or other molecules, such as bio-stimulants, is a common agronomic practice for all marketed maize seeds, to improve both their germinability and the vigor of seedlings.

Few chemical compounds are registered to control fungal diseases on maize and other cereals; moreover, at the same time, the occurrence of resistance to fungicides in fungal populations can reduce the effectiveness of the molecules widely used for seed treatments. Both integrated pest management guidelines and environmental sustainability policies encourage researchers to develop newer eco-friendly strategies to control both fungal species and their mycotoxin accumulation, in order to reduce chemical treatments and, consequently, their negative environmental impact.

The word plasma refers to ionized gases in thermodynamic equilibrium conditions (thermal) or far from them (cold). Technologies based on cold plasmas, in particular, impact our everyday life with so many surface-modification processes and commercial products in strategic areas such as microelectronics, semiconductors, polymers, and biomaterials, just to mention a few [20]. Plasma deposition, treatment, and etching processes are utilized for altering the surface properties of materials with an added or ablated thin layer, usually thinner than one micron, in the range of tens to hundreds of nanometers. Since surface-modification plasma techniques use a minimum amount of reagents and no solvents, they are considered eco-friendly. Due to their low thermal impact, also, they allow the modification of the surface of thermo-labile substrates with no changes to their bulk properties. In the last two decades, cold plasma technologies have impacted also life sciences, such as medicine, agriculture, and food science, with newer approaches and processes in anti-cancer therapies, as well as in the decontamination of wounds, field water, seeds, and fresh food [20,21,22].

Cold plasma technologies are being widely tested on seeds to enhance the germination rate and plant growth [23,24,25,26,27]. They have been also applied for inactivating pathogenic and spoilage microorganisms in food [28,29,30]. All proposed applications of cold plasma technology for agriculture, including water treatments, are presented in Puac et al. [31]. 

We carried out a preliminary study applying a fungicide on maize seeds using plasma technology, by embedding it with a coating plasma-deposited directly on the seeds, for better investigation of the potentiality of this technology in controlling fungal infections and reducing the use of fungicides in agriculture [32]. Among marketed fungicides, Masiello et al. reported that the de-methylation inhibitor prothioconazole was the most active molecule in vitro against the most important mycotoxigenic fungi of maize and confirmed its effectiveness in reducing *F. graminearum* and *F. proliferatum* contamination of maize plants in the field [33]. Based on this knowledge, Lo Porto et al. [32] developed a plasma-deposition approach to apply prothioconazole directly and only at the surface of the maize seeds, to enhance the effectiveness of the fungicide, to limit its used quantity, and to avoid its dispersion in the soil. Indeed, this approach is original so far in the literature, since plasma treatments of seeds, generally in the form of ablation and hydrophilization of their surfaces, are generally tested for direct decontamination or for inducing faster germination, but not plasma deposition. Most surface modification (etching, grafting, and deposition) cold plasma processes have been developed at low (10–1000 mTorr) pressure. In the last decades, however, for several reasons, processes at atmospheric pressure have been developed too [34,35]. Two low pressure plasma-deposition processes were used by Lo Porto et al. [32] to develop first a thin wettable layer around the seeds, where the prothioconazole could be sprayed and remain, and then a hydrophobic layer aimed to embed the fungicide, and repristinate the natural hydrophobicity of the seed surface. The effective total thickness of the two layers is in the order of hundreds of nanometers. This approach allowed the effective plasma-assisted immobilization of the fungicide on the maize seeds without interference with their water absorption and germination, and the reduction of the effective dose of the fungicide. In order to test the full applicability of such an approach to control infection from external sources of both *F. graminearum* and *F. proliferatum*, and determine if they could be systemically transmitted, we carried out experiments both in vitro and in planta with the following aims: 

To evaluate the possible increased effectiveness of prothioconazole embedded in close contact with the seeds, with a plasma-deposition protocol, against *F. graminearum* and *F. proliferatum*; 

To test the effects of the plasma-deposition protocol on seed germination and the transmission of *F. graminearum* and *F. proliferatum* infection from maize seeds; 

To evaluate the possible systemic transmission of *F. graminearum* and *F. proliferatum* from seeds to seedlings.

## 2. Results

### 2.1. In Vitro Effect of Plasma Coating and Prothioconazole Application, Alone or in Combination, against Fusarium Species 

**Figure 1:**
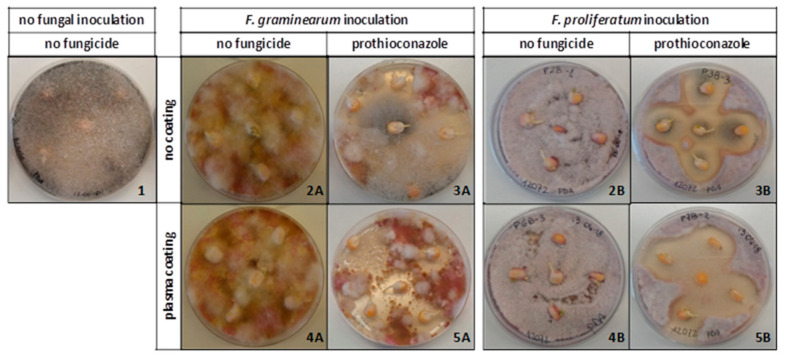
Maize seed contamination evaluated after 3 days, on different Theses, inoculated and not inoculated with *Fusarium*, coated and not by means of plasma deposition, treated and not with prothioconazole. The thesis number is shown at the bottom right of each picture.

*Fusarium proliferatum* was detected in 7% of seeds in the control (Thesis 1), while *F. graminearum* was not found. Moreover, an unexpected heavy contamination by *Rhizopus* occurred in Theses 1, 3A, and 3B, with contamination values of 100%, 13%, and 20%, respectively (Figure 1). 

After 3 days of incubation, *Fusarium* strains had colonized the entire surface of PDA plates. For both *Fusarium* species, the treatment with no plasma coating and no prothioconazole (Theses 2A and 2B), and the treatment with only plasma coating and no fungicide (Theses 4A and 4B) were completely (100%) colonized by *Fusarium*; after 10 days of incubation, the seeds were completely colonized by *Fusarium* mycelium. In the theses treated with prothioconazole only, the fungicide showed a good efficacy to reduce fungal colonization (Theses 3A and 3B), and the seeds remained healthy and all germinated. In particular, around each seed, an inhibition halo of about 2.5 cm of diameter was observed after 10 days of incubation. Indeed, the best activity to control both *Fusarium* species was obtained in Theses 5A and 5B, where the seeds treated with the prothioconazole embedded in the plasma-deposited coating were grown. These seeds resulted in being completely free of *Fusarium* mycelium, and around each of the seeds an inhibition halo with a diameter of 3.5 cm was observed. 

**Figure 2:**
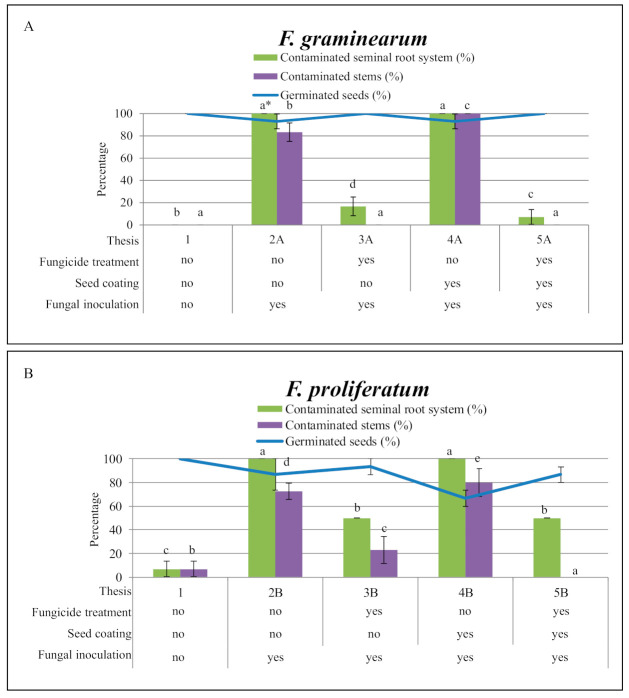
Percentage of seed germination and fungal contamination in maize seedlings grown in Petri dishes containing synthetic medium, after different and combined treatments (fungicide application, plasma coating, fungal inoculation). Two separated experiments were carried out for *F. graminearum* (**A**) and *F. proliferatum* (**B**). * Values with different letters are statistically different with *p* < 0.05 as determined by one-way analysis of variance (ANOVA) followed by the Tukey test.

Data on seed germination and fungal contamination assessed for each thesis in the two experiments for *F. graminearum* (A) and *F. proliferatum* (B) are reported in Figure 2.

#### 2.1.1. Seed Germination 

Seed germination rate was assessed 7 days after seed sowing. All seeds of the control (Thesis 1) germinated (100%). 

*Fusarium graminearum*. Seeds inoculated with *F. graminearum* (Figure 2A) showed 100% germination when prothioconazole was applied alone (Thesis 3A) or in combination with the plasma-deposited coating (Thesis 5A), and 93% for the seeds that were not treated with the fungicide (Thesis 2A), in presence of the plasma-deposited bilayer (Thesis 4A). We calculated the standard error (SE) values for all theses. Theses 2A and 5A had SE values of 6.7.

*Fusarium proliferatum*. In the theses inoculated with the conidial suspension of *F. proliferatum* (Figure 2B), when prothioconazole was applied alone or embedded in the plasma-deposited bilayer, seed germination rates of 93% (Thesis 3B; SE 6.7) and 87% (Thesis 5B; SE 6.7) were measured, respectively. The lowest germination values of 87% (Thesis 2B; SE 13.3) and 67% (Thesis 4B; SE 6.7) were observed in the case of seeds untreated or treated only with the plasma coating.

#### 2.1.2. Transmission of the Fungi to Roots and Stems 

In this experiment, the capability of the two *Fusarium* species to colonize seedling tissues from the seeds was evaluated. 

Control. In the control (Thesis 1), *F. graminearum* was not detected in any root and seedling tissues, while *F. proliferatum* was detected in 7% of both seminal root systems and stem tissues (Figure 2). 

*Fusarium graminearum*.

Roots. For *F. graminearum*, the seminal root system of seedlings of both theses untreated with prothioconazole was found completely colonized, showing contamination values of 100%. In the presence of prothioconazole instead, used alone or embedded in the bilayer coating, the colonization of the seminal root system by the pathogen had poor results (17 and 7%, respectively). In particular, a statistically significant difference between Theses 3A (SE 8.3) and 5A (SE 6.7) was observed, in favor of the fungicide embedded in the coatings. 

Stems. For *F. graminearum*, the stems of seedlings of both theses untreated with prothioconazole were highly colonized and showed contamination values of 100% (Thesis 4A) and 83% for the stems of Thesis 2A (SE 8.3). On the contrary, in theses treated with prothioconazole, used alone or embedded in the bilayer coating, there was not colonization of the stems by *F. graminearum*. 

*Fusarium proliferatum*.

Roots. The seminal root systems of the seedlings with no prothioconazole treatment (Theses 2B and 4B) were both totally colonized by *F. proliferatum* (100%), with no statistical difference between each other. Instead, contamination values of 50% were detected on the seedling root systems for the seeds treated with prothioconazole (Theses 3B and 5B, values of SE were 0 for both theses), combined or not with the plasma coating. 

Stems. *Fusarium proliferatum* was found to be able to colonize the stems moving from the seeds to stem tissues of the seedlings. In the theses untreated with prothioconazole, stem contamination values of 73% were found in Thesis 2B (SE 6.7) and of 80% in Thesis 4B (SE 11.5). Seedling stems of seeds exposed to prothioconazole alone, Thesis 3B, were found poorly colonized (23%, SE 11.7); in addition, *F. proliferatum* was not detected in the stems of the seedlings resulting from seeds treated with prothioconazole embedded in the plasma bilayer (Thesis 5B). 

### 2.2. In Planta Effects of Plasma Coating and Prothioconazole Application

**Figure 3:**
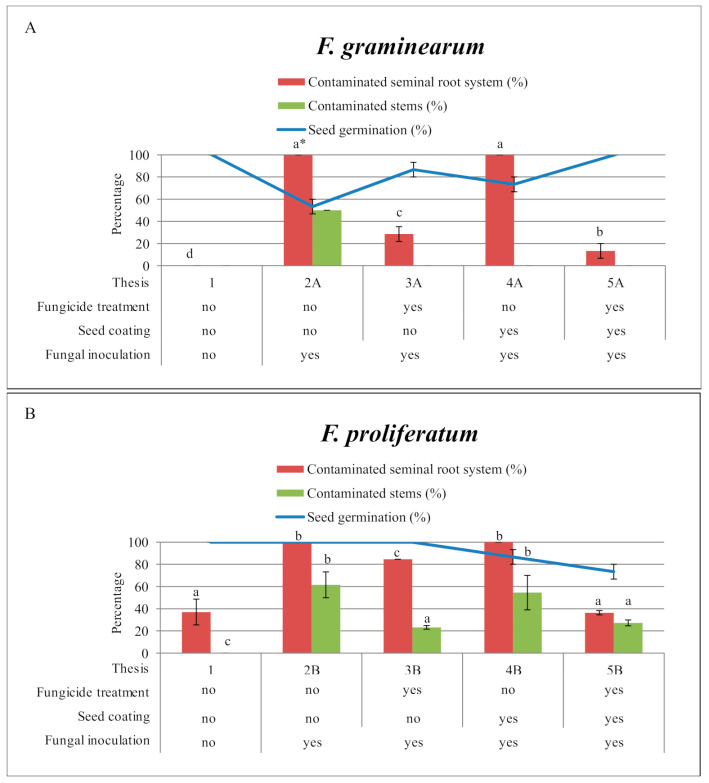
Percentage of seed germination and fungal contamination in maize seedlings grown in pots containing soil, after different and combined treatments (fungicide application, seed coating, fungal inoculation). Two separated experiments were carried out for *F. graminearum* (**A**) and *F. proliferatum* (**B**). * Values with different letters are statistically different with *p* < 0.05 as determined by one-way analysis of variance (ANOVA) followed by the Tukey test.

The in-planta results are summarized in Figure 3. The efficacy of the plasma-based coating combined with the application of prothioconazole directly on the maize seeds was tested on maize seedlings grown in pots containing soil, to mimic field growth conditions.

#### 2.2.1. Seed Germination 

Seed germination rate, shown in Figure 3, was measured 7 days after the emergence of the seedlings. In the control (Thesis 1), all seeds germinated (100%). 

*Fusarium graminearum*. In the experiment with *F. graminearum* (Figure 3A), for seeds not treated with prothioconazole nor plasma (Thesis 2A) or treated only with the plasma coating (4A), the germination of seeds was found to be decreased, with values of 53% (SE 6.7) and 73% (SE 6.7), respectively. For seeds treated with prothioconazole, alone (Thesis 3A) or in combination with the plasma deposition (Thesis 5A), germination rates of 87% (SE 6.7) and 100% were measured, respectively. 

*Fusarium proliferatum*. In the experiment with *F. proliferatum* (Figure 3B), seed germination of 100% was measured for seeds untreated or treated with prothioconazole, without plasma coating (Thesis 2B and 3B). A decreased germination rate was observed in both theses where the seeds were plasma coated, alone or in combination with embedded prothioconazole, with germination values of 87% (Thesis 4B; SE 6.7) and 73% (Thesis 5B; SE 6.7), respectively.

#### 2.2.2. Colonization of Roots and Stems 

*Fusarium graminearum*.

Roots. In the experiment with *F. graminearum* (Figure 3A), for seeds not treated with prothioconazole, 100% of the root system was found colonized by the fungi. Statistical differences were not observed between inoculated control theses and theses treated only with the plasma coating. For seeds treated with prothioconazole, alone or in combination with the plasma-deposited bilayer, statistically different root system contamination values of 29% (Thesis 3A; SE 6.7) and 13% (Thesis 5A; SE 6.7) were found, respectively. *Fusarium graminearum* was absent in the root of seeds that were not inoculated. 

Stems. With regard to stem colonization, fungal contamination was observed only in Thesis 2A, seeds with no prothioconazole nor plasma coating, but inoculated with *F. graminearum*, with a value of 50% (SE value was 0). Although inoculated with *F. graminearum*, Theses 3A, 4A, and 5A did not show any fungal contamination.

*Fusarium proliferatum*. 

Roots. In the experiment with *F. proliferatum* (Figure 3B), *F. proliferatum* was detected also in the not inoculated Thesis 1, with the value of 37% in the root system (Thesis 1; SE 11.5). Theses 2B and 4B, both not exposed to prothioconazole, revealed that 100% of the root system was contaminated. Theses 3B and 5B, where prothioconazole was used, alone or in combination with the plasma coating, showed root system contamination values of 85% (Thesis 3B; SE 0) and 36% (Thesis 5B; SE 1.9), respectively. Interestingly, Theses 1 (not inoculated seeds) and 5B, were statistically grouped in the same homogeneous group, demonstrating the strong effect of prothioconazole combined with the plasma coating on the seeds against *Fusarium* colonization. 

Stems. With respect to stem contamination, 62% of stem contamination was recorded for untreated seeds (Thesis 2B; SE 11.7), while in seeds protected by the plasma coating (Thesis 4B), 55% (SE 15.5) of stems were colonized by *F. proliferatum*. No statistical difference was observed between Theses 2A and 4A. Theses 3B and 5B, treated with prothioconazole, alone or in combination with the plasma bilayer, had values of 23% (SE 1.7) and 27% (SE 2.7) of stems colonized by *F. proliferatum*, respectively. 

#### 2.2.3. Disease Symptoms on Seedlings 

**Figure 4:**
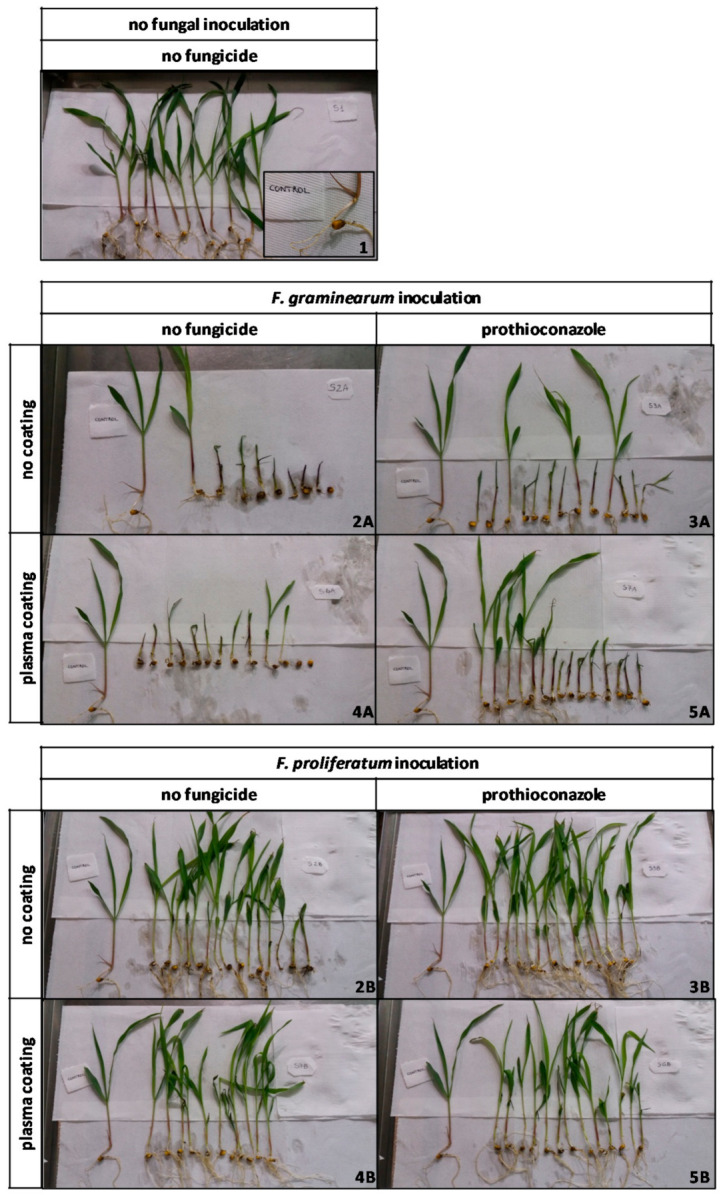
Ten-day-old maize seedlings, unrooted after growing in phytotron. The theses number, representing the different combined treatments, are reported at bottom right of each picture, compared to the control thesis, untreated and not inoculated with *Fusarium* species.

The general appearance of the seedlings was evaluated 10 days after their emergence. The plants of the control (Thesis 1), as well as those inoculated with *F. proliferatum* (Theses 2B, 3B, 4B, 5B), appeared vigorous, intensely green colored, 10–15 cm high, and with no evident symptoms of *Fusarium* infection. The plants inoculated with *F. graminearum,* instead, appeared pale green, 3–8 cm high, and with red mycelium present on the stems (Figure 4).

## 3. Discussion

*Fusarium* species colonize maize plants through different infection pathways, being infected seeds, crop residues, and soil primary inoculum sources for infection of roots, seedlings, and stems. The data reported in this study provide evidence that *F. graminearum* and *F. proliferatum* transmission from maize seeds to seedlings in effect occurs. In all theses inoculated with both species, maize stems resulted in being colonized by both fungi. Seed transmission of *F. graminearum* is well known and widely reported only for wheat [36,37], while for maize, only few recent studies exist [38]. Similarly, systemic infection of *F. proliferatum* in maize has been poorly investigated, although a disease cycle similar to *F. verticillioides* was hypothesized by Munkvold and Desjardins [8]. The *Fusarium* contamination on maize in the early growth stage leads to seedling decay and reduced crop stands. Moreover, systemic infection during the early growth stage may remain latent, causing disease when suitable conditions occur, and the mycotoxins eventually produced at this stage could translocate from stems to ears and accumulate in the kernels. Therefore, coating of the seed can reduce these risks.

Some authors reported that seed treatments with chemicals can effectively suppress or reduce seed-borne pathogens in maize [39,40], thus reducing crown and stalk rot, while enhancing photosynthesis and improving the vigor of maize plants [41]. However, most data regard *F. verticillioides* [42]. We reported a high capability of prothioconazole in inhibiting *F. verticillioides*, *F. graminearum*, and *F. proliferatum* in vitro and in field experiments, with respect to other fungicides [33]. Furthermore, prothioconazole encapsulated in the plasma-deposited bilayer has been reported to successfully reduce *F. graminearum* infection in maize seedlings [32]. The current technologies for seed coating provide several advantages, such as the containment of the chemical compounds in confined spaces, and the possibility to reduce the dose of fungicides to control fungal development, with a consequent decreased release of chemicals into the environment. To date, the main application of plasma technology is using cold plasmas at atmospheric pressure deactivating fungal spores and bacteria, therefore improving seed germination and seedling growth to obtain a higher vigor of the plants. 

The present innovative work demonstrates the efficacy of embedding prothioconazole sandwiched in a plasma-deposited bilayer coating directly at the surface of the seeds in reducing *F. graminearum* and *F. proliferatum* infection of seeds and seedlings. At the same time, we have evaluated the effect on maize seed germination. In the experimental trial in phytotron, after inoculation of *F. graminearum*, a slightly higher seed germination rate was observed in all theses with the plasma-coated maize seeds. On the contrary, plasma-coated seeds inoculated with *F. proliferatum* showed a decreased germination rate. 

The plasma coating alone decreased the level of maize stems colonized by *F. graminearum* from 50% to 0%, while the colonization by *F. proliferatum* was reduced to a lower extent, from 62% to 55%. On the other hand, the stem colonization by *F. proliferatum* in seeds treated with prothioconazole was not affected by the presence of the plasma coating. On the contrary, *F. graminearum* growth in stems was found totally inhibited after treatment with prothioconazole alone or encapsulated in a plasma coating. Seed infection of 100% for both *F. graminearum* and *F. proliferatum* was observed in phytotron trials, except when seeds were treated with prothioconazole alone or encapsulated in a plasma coating. The infection value of seeds treated with prothioconazole decrease to 29% for *F. graminearum* and to 85% for *F. proliferatum*. The efficacy of prothioconazole against *F. graminearum* and *F. proliferatum* was previously proven in the field by Masiello et al. [33]. Our present results obtained with prothioconazole encapsulated in a plasma coating show the reduction of seed infection to 13% for *F. graminearum* and to 36% for *F. proliferatum*.

Zahoranova et al. [29] reported that the use of cold atmospheric pressure plasma treatments in air on maize seeds allowed a complete devitalization of the native microbiota at the surface of the seeds, showing a consequent potential reduction of the use of hazardous chemicals [29]. Interestingly, a further study has reported the potential of cold plasma processes on roasted coffee to both improve fungal control and induce mycotoxin degradation [28]. However, several studies on the use of cold plasma processes to inactivate toxigenic fungi and detoxify different kinds of seeds (mostly nuts) have shown a dramatic variability of plasma efficacy [43,44,45], perhaps related to the influence of many parameters such as shape, surface, and size of the seeds, as well as by the sensitivity of each fungal species target. Finally, the homogeneity of the treatments around each single seed clearly plays a role also.

## 4. Materials and Methods

### 4.1. Maize Kernels, Fungicide Application, and Plasma-Deposited Coatings

Certified uncoated maize seeds of the hybrid variety Marano 0501, marketed by Società Italiana Sementi (SIS Spa, Bologna, Italy), were selected for our experiments.

One hundred randomly selected seeds were tested to evaluate their natural endophytic fungal contamination. Ten seeds per plate, surface sterilized with a 2% sodium hypochlorite solution and washed twice with sterile distilled water, were placed on potato dextrose agar (PDA) medium. After 5 days of incubation at 25 °C, the fungal colonies originating from infected seeds were counted and morphologically identified. The fungal contamination was expressed as a percentage of contaminated seeds of total tested seeds. The seeds resulted in contamination by *F. proliferatum* at a low level (2%), while *F. graminearum* was not detected. Prothioconazole, a systemic fungicide that interferes with fungal sterol biosynthesis, widely used to control *Fusarium* species on cereal seeds or with foliar applications, was applied at the surface of the seeds as described below. 

Part of the maize seeds were treated with the deposition of a plasma-based bilayer coating, as described by Lo Porto et al. [32]. A home-made stainless steel parallel-plate capacitive-coupled RF (13.56 MHz)-driven low pressure plasma reactor was used to deposit a two-layer coating at the surface of maize seeds. Prothioconazole was delivered at the surface of the seeds by spraying 2 g of a water solution containing 3 μL of commercial formulate PROLINE (containing 25% active ingredient; Bayer Crop Science, Leverkusen, Germany) under shaking conditions, with 30 seeds positioned in a Petri dish. Detail on the reactor and on the deposition processes are published in Lo Porto et al. [32]. The seeds were positioned on the lower, grounded electrode of the reactor, and treated in the following experimental conditions:

Inner coating: C_2_H_4_ (2.5 standard cc/min, sccm) and CO_2_ (5 sccm) were used to feed glow discharges of 20 min duration. The inner coating of about 180 nm of thickness resulted at the surface of the seeds, characterized by a water contact angle (WCA) of 88 ± 3°, slightly lower than that of the seeds with 103 ± 11°. This hydrophilic coating was deposited to enhance the wettability of the seed to the prothioconazole solution. 

Outer coating: C_2_H_4_ (7.5 sccm) alone was used to feed glow discharges of 20 min duration, after the deposition of the inner coating and, for certain seed aliquots, after the administration of the prothioconazole solution. The outer coating of about 800 nm of thickness resulted onto the seeds, characterized by a WCA of 106 ± 3°, similar to the hydrophobic pristine value of the untreated seeds.

The seeds treated with the two coatings are often defined as plasma-coated in the whole paper. When applied, prothioconazole is embedded between the two plasma-deposited coatings, by spraying of the above-described solution.

### 4.2. Fungal Strains and Preparation of Inoculum

One strain each for *F. graminearum* and *F. proliferatum* was selected from the ITEM Collection of ISPA-CNR (www.ispa.cnr.it/Collection). The *F. graminearum* ITEM 126 is a highly DON-producing strain [37]; the *F. proliferatum* strain ITEM 12,072 is a high FBs-producing strain. Both *Fusarium* strains were used in a previous study to test their sensitivity to different fungicides, including prothioconazole [33]. For each *Fusarium* strain, a conidial suspension was prepared by scrapping with a sterilized loop the mycelium of 7-day-old colonies dissolved in distilled sterilized water containing 0.01% of Tween 20. The conidial suspension was adjusted to 1 × 10^5^ conidia/mL for both *F. graminearum* ITEM 126 and *F. proliferatum* ITEM 12072, using the Thoma cell counting chamber. At the same time, five plugs of each 7-day-old colony were added to 200 g sterilized rice and incubated at 25 °C for 14 days. This colonized rice was used as inoculum for in planta experimental trial. After incubation, *Fusarium* colonized rice was gently ground and mixed with sterile river sand in a 1:1 ratio.

### 4.3. In Vitro Experimental Assays

Two different trials were carried out for *F. graminearum* and *F. proliferatum*. For each species, four different theses inoculated by the fungal strains were set up and numbers from 2 to 5 were assigned to each thesis, as described in Table 1: thesis with no plasma coating or fungicide treatment; thesis with no plasma coating and fungicide treatment; thesis with plasma coating and no fungicide treatment; thesis with plasma coating and fungicide treatment.

For each thesis, fifteen maize seeds were placed on PDA amended with pentachloronitrobenzene (PCNB). All four theses were inoculated with the conidial suspension: theses inoculated with *F. graminearum* are denoted by the letter A; theses inoculated with *F. proliferatum* are denoted by the letter B. The plasma-deposited bilayer coating was utilized on the seeds of Theses 4 and 5; prothioconazole was applied to Theses 3 and 5 (Table 1), as described earlier. All theses were compared with a negative control (Thesis 1), in which seeds were not inoculated with the conidial suspension, and not exposed to any of the plasma and prothioconazole treatments.

In all the inoculated theses, 100 µL of conidial suspension was spread on the PDA medium of each Petri dish and 5 maize seeds were then placed in each plate and incubated at 25 °C under fluorescent light (12 h of photoperiod).

The effect of each treatment in inhibiting the fungal colonization of the seeds was evaluated after 3, 5, 7 and 10 days of incubation. The effects of the treatment on seed germination and on the capability to control maize seedling colonization were also evaluated at the same time. All considered values are expressed in percentages. 

To evaluate the systemic colonization of maize seedlings by *F. graminearum* and *F. proliferatum*, all seedlings were collected after 10 days, surface sterilized with a 2% sodium hypochlorite solution, washed twice with sterile distilled water, and dried on a sterile filter paper in a laminar flow hood. For each seedling, three small pieces were cut from the seminal root system and from the seedling stem and placed on Petri dishes containing PDA amended with PCNB. Petri dishes were incubated at 25 °C under fluorescent light (12 h of photoperiod). After 6 days of incubation, the contamination level of the two different seedling portions was expressed as a percentage of contaminated tissues on total plated parts.

### 4.4. In Planta Experimental Trials in Phytotron

To test the efficacy of the plasma-based coating embedding the fungicide prothioconazole against *F. graminearum* and *F. proliferatum* in planta, a similar approach was used to that described in Table 1.

For each thesis, 3 pots with max capacity of 2.7 L (as suggested by the manufacturer) and size of 14 × 14 × 16 cm^3^ were filled with 600 g of sterilized soil and wet with 250 mL of sterilized water. Thirty grams of *Fusarium*-colonized rice mixed with sterile river sand was distributed at the surface of each pot of theses 2, 3, 4 and 5, in a 1:20 ratio of inoculum/soil. Thirty grams of sterile river sand without *Fusarium*-colonized rice was distributed on the control Thesis 1. The pots were covered with a plastic film and placed in a growth chamber to favor the growth of the fungal inoculum on the soil. After 24 h, 15 maize seeds (5 per pot) for each thesis (Table 1) were placed on the surface of the inoculum/sand mixture and covered with 1 cm of sterile soil. The soil was wet with 150 mL of sterile water and the pots, covered again with the plastic film, were kept for 4 days in the growth chamber at 23 °C with a 12 h light/darkness photoperiod. The pots were then moved to a phytotron, to allow the growth of maize plants in controlled conditions: 23 °C, 85% of relative humidity, 50 lumen, and 12 h light/darkness photoperiod.

The rate of germination and the general growth of seedlings, in terms of height, vigor, color, and general appearance, were evaluated together with disease symptoms 3, 5, 7 and 10 days after the emergence of the seedlings Seed germination was evaluated by observing the number of seedlings emerged from the total number of seeds sowed, expressed in percentages. 

The symptoms of *Fusarium* infection that were evaluated included the wilting and reddening of the seedlings and the presence of aerial mycelium on stems.

To evaluate the systemic colonization of maize seedlings by *F. graminearum* and *F. proliferatum*, the seedlings of each thesis were uprooted and collected in paper bags after 10 days. After removing the soil from the roots, the whole seedlings were surface sterilized with a 2% sodium hypochlorite solution, washed twice with sterile distilled water, and dried on sterile filter paper under a flow hood. From each seedling, small pieces of root and stem were cut of about 5 mm, transferred to Petri dishes containing PDA amended with PCNB, and incubated at 25 °C. The colonization of root seeds and aerial portions of each thesis, expressed as a percentage of colonized tissues on total analyzed portions, was checked after 6 days of incubation.

### 4.5. Statistical Analyses

The SE values for all theses were calculated. Statistical analyses of data were performed using the statistical package STATISTICA v. 6.0 software (StatSoft, Tulsa, OK, USA). A one-way ANOVA was used to test the hypothesis of treatment differentiation with respect to *Fusarium* of both seminal root systems and stems. The means were then compared using Tukey’s honestly significant difference (HSD) test, with a significance level (*p*) of 0.05.

## 5. Conclusions

The dramatic reduction of the infected seeds suggests the fungicide coupled with the plasma coating was the most efficient approach for the reduction of contamination and highlights how promising this could be for maize and other seeds. Very likely, the stronger protection effect can be explained by the longer permanence of the fungicide in close contact with the seeds during growth, with the slower release of the molecules in the soil through the bilayer coating. Furthermore, the presence of the coating probably protects the seeds by preventing the infection from the soil and by extending the period of time during which the fungicide is available. Another important achievement of this study is the potential strong reduction of the amount of fungicide used for seed protection. The great attention paid to the environment by the recent Green Deal EU program has clearly defined, as the main object, a significant reduction of chemicals in the next 30 years in the whole EU. Our findings suggest that the application of plasma technology not only offers a potential great improvement for the efficient use of resources in the food industry but would also be a key achievement for improving food safety policy, towards the direction of a more sustainable approach in agriculture.

## Figures and Tables

**Table 1:** Description of theses used for experimental assays in vitro and in planta.

Theses	Fungal Inoculation	Seed Coating	Fungicide Application
1	not inoculated	No	No
2A	*F. graminearum*	No	No
3A		No	Prothioconazole
4A		Plasma coating	No
5A		Plasma coating	Prothioconazole
2B	*F. proliferatum*	No	No
3B		No	Prothioconazole
4B		Plasma coating	No
5B		Plasma coating	Prothioconazole

## Data Availability

The data is contained within this article.
